# Aerodynamic characterisation of porous fairings: pressure drop and Laser Doppler Velocimetry measurements

**DOI:** 10.1038/s41597-023-01934-5

**Published:** 2023-01-19

**Authors:** Fabien Méry, Delphine Sebbane

**Affiliations:** ONERA, Université Fédérale de Toulouse, Toulouse, France

**Keywords:** Aerospace engineering, Fluid dynamics

## Abstract

Wind tunnel measurements of pressure drop and steady and unsteady velocity field of a flow through fairing samples are described. 10 samples have been tested in pressure drop among which the velocity fields of 3 samples have been characterized by means of laser Doppler velocimetry. The samples are perforated plates, wiremesh plates or complex 3D geometries resulting from additive manufacturing methods. The Reynolds number of the experiments ranges from 55 000 to 117 000.

## Background & Summary

Aviation has become a mass transportation industry, and all prospective studies foresee growth in this sector. Among the challenges, noise in the vicinity of airports has gone from a marginal annoyance to a real public health concern. To address this problem, as well as others such as fuel consumption, aircraft manufacturers are considering radically new aircraft architectures that could enter service quickly. In the meantime, however, the noise of traditional aircraft must be reduced significantly. Aircraft noise, during takeoff and landing, results primarily from a combination of (i) engine noise, which is generated by the fan and jet, and (ii) airframe noise, primarily due to the landing gear (LG) and high lift devices (HLD), the latter including slats and trailing edge flaps, which are deployed at low speeds to increase lift^1^. During takeoff, engine noise remains dominant, while on approach and landing, engines operate at low speeds (typically 50% of N1), and airframe noise becomes a significant contributor, especially for newer aircraft equipped with latest generation turbofans. Its mitigation is therefore of primary interest^2,3^. However, due to the strong integration constraints imposed by other disciplines than acoustics on components such as LGs and HLDs, the development of noise reduction technologies (NRT) on these airframe components has been limited. This lack of breakthroughs is also due to the complexity of flow physics, and thus our still limited knowledge of airframe noise generation mechanisms. The noise of the landing gear, slats and flaps has been studied on a real and reduced scale, mainly on the basis of experimental means^4–6^. The maturity of numerical simulations now allows to study the mechanisms of the noise sources on various complex configurations^7,8^. Moreover, numerical simulation methods can be sufficiently accurate to predict the noise generated by such configurations. In order to take the next step in the maturity of numerical prediction, these NRTs must be accurately evaluated and modeled. Experimental data based on academic configurations are therefore needed to validate the new tools and numerical models.

One promising NRT is the use of a fairing in front of the landing gear to reduce the noise of this system^9^. The present study aims at collecting an experimental database (pressure drop and turbulence characteristics) of several fairing solutions in order to have validation test cases for CFD simulation and thus develop new models for such complex geometries. The fairing samples are thus tested on the “Acoustic and Aerothermal Bench” (B2A), by measuring the pressure drop of each sample and the flow field by Laser Doppler Velocimetry (LDV). The experimental methodology will be presented first. The database will then be described. Some technical validations will also be proposed on the basis of a comparison with the literature.

## Methods

### Test rig, Set-up and instrumentation

These experiments are intended to allow the validation of models and numerical simulation codes. In this perspective, Oberkampf *et al*.^10^ proposes some guidelines to ensure that the quality and description of the experiments are as complete as possible and proposed a rating level (from 0 to 3) for wind tunnel tests: the model validation experiment completeness (MVEC). We guarantee here a MVEC level of 2 regarding the available informations in this data descriptor.

**Fig. 1:**
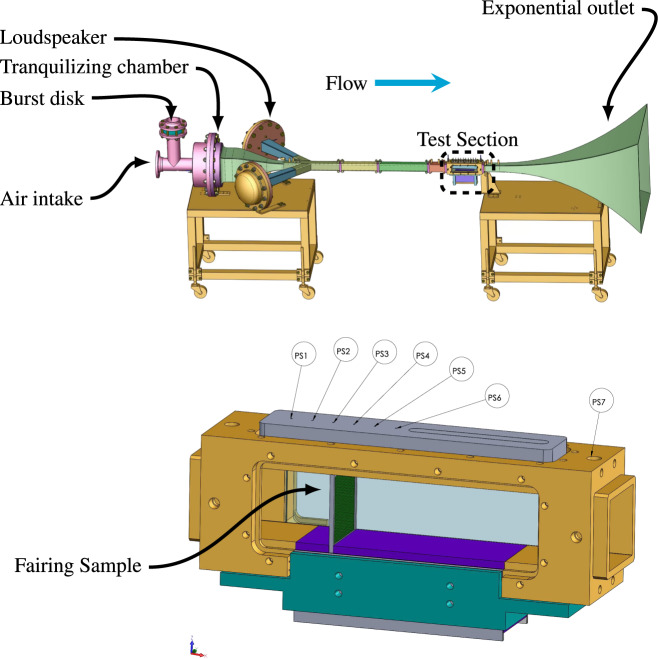
B2A setup for INVENTOR activity. Overall view of B2A windtunnel. Overall length: 3880 mm and Magnified view of B2A INVENTOR Test section (50 mm × 50 mm). The fairing sample is located inside the test section, perpendicular to the flow. Overall size of the test section: 280 mm.

The aeroacoustic test bench (B2A)^11^ at ONERA is made of a stainless steel tube of section 50 × 50 mm; its total length is about 3880 mm. The distance between the convergent and the test section is 1300 mm which ensures a fully developped flow. A 0.2-m-long test section is equipped with two silica windows for optical access. The termination is equipped with a quasi-anechoic outlet, leading to an upstream reflection coefficient smaller than 0.2 for frequencies higher than 500 Hz. A mean flow with a bulk Mach number *M*_*b*_ up to 0.5 can be provided. The static flow temperature can be accurately regulated from the ambient temperature up to 300 °C. In the test section, this flow shows fully developed turbulent boundary layers, with a turbulence rate of a few percent at the center of the test section. As shown in Figure 1, flow propagates from left to right. The test bench is connected to a pressurized air tank. A mass flow control valve can regulate the mass flow with a high accuracy (less than 1% error on the imposed mass flow rate). This mass flow rate is measured with a flow meter (Rosemount 485 Annubar) installed on the mass flow control valve. It ranges from 50 g/s up to 500 g/s. The bulk Mach number can be thus derived from the mass flow definition.

Figure 1 presents an overview of the experimental setup. The fairing sample is placed across the test section and covers all its cross-sectional area. Static pressure taps are available on the top of the test section upstream and downstream the sample. Table 1 gives the position of the pressure tabs (PS1 is the reference). The sample is placed 37.5 mm downstream the PS1 pressure tap. The static pressures are monitored and acquired using the SVMTec differential pressure scanner. The scanner has a 1.250 kPa range with an uncertainty of max ±0.1% of the full scale span (non-linearity and hysteresis). The sampling rate is 10 Hz and 200 samples are acquired. The reference pressure tap PS1 is also measured by a 45 Psi Digiquartz absolute pressure sensor in order to have the absolute static pressure upstream to the sample (uncertainty of 0.01% of the full scale span) The sampling rate is 4 Hz and 80 samples are acquired. When the SVMtec scanner is out of range for high pressure drop samples, only the Digiquartz sensor is used on all the pressure taps.

**Table 1:** Pressure taps position.

Pressure label	PS1	PS2	PS3	PS4	PS5	PS6	PS7
Streamwise position [mm]	0	15	30	45	60	75	215

A two-component fringe-mode LDV allows the measurement of the axial U and vertical W velocity components in almost the entire volume of the test section^12^ mounted on a 3-axis traverse system was used in forward-scattering configuration to maximize signal to noise ratios and data rates. This measurement system was composed of a laser emitting green (514.5 nm) and blue (488 nm) wavelengths. The two pairs of beams were issued by a DANTEC 55X emitting head equipped with a 240 mm focal lens. The fringe spacing for the green beam is 3.7232210^−6^ ± 1.7729110^−8^ m and the fringe spacing for the blue beam is 3.5425910^−6^ ± 1.5953410^−8^ m. The crossing angles are measured and used as an input for the post-processing of the signals. The transformation matrix as an accuracy better than 1%. Signals were processed by an IFA755 burst spectrum analyzer and velocity statistics. The emitting optics produce an elliptical measurement volume whose minor axis can be as small as 50 microns and the major axis is 0.7 mm. Flow is seeded with amorphous silica particles, injected upstream of a flow rectifier and the convergent module, in order to ensure an homogeneous distribution. This choice of particle seeding over paraffin- or oil-based seeding is initially motivated by its weak deposit over time on the test cell windows. According to the manufacturer, the mean primary particle size is about 20 nm, with aggregates up to 0.2 microns. Considering the largest particles with a conservative maximum diameter of 0.5 microns, the minimum frequency response in our test conditions was estimated to be around 15 kHz with less than 1% slip, and the maximum Stokes number to be around 0.025, lower than 0.1.

The LDV signal is unevenly sampled due to the random arrival of particles in the measurement volume.

A reconstruction method^11,12^ is used to resample the raw data at a constant rate. Signals are processed using the in-house ONERA software, ASSA^13^. A minimum sampling rate of *f*_*m*_ = 15 kHz measurements per second is ensured, for each velocity component, and more than 200,000 samples are acquired so that statistical convergence of the mean velocity is largely ensured, only coincident signals were accepted. The U and W components are measured for 4 (X,Y) planes. Each planes are composed of a regular grid of 6 × 6 points (36 points). Each grid dimension is 25 mm × 25 mm centered in the test section. The first plane is measured upstream the sample (12 mm upstream the sample) and the 3 other planes are positioned downstream the sample (35 mm, 81 mm and 128 mm).

### Sample description

**Fig. 2:**
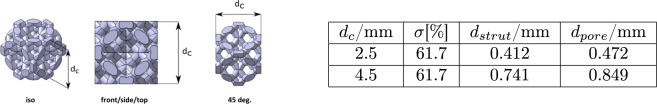
Diamond grid model and characteristics.

The samples are adapted to fit in the B2A test section: the samples cover the whole cross-test section surface. Tables 2–4 show the tested samples with their label and the measurements performed on each one. The Sheet with Cylindrical Perforations (SCP) **D***a***T***b***e***c* types are perforated panels: *D* = *a* represents the hole diameter in mm, *T* = *b* represents the distance between holes in mm and *e* = *c* the material thickness in mm. The angle Φ represents the angle of the perforations (one sample only with this feature). The WireMesh (WM) is a classical grid (LT type^14^), it is an interwoven round wires. The wire diameter is *d* and *M* is the mesh width: in the present case, *M* = 5 mm and *d* = 1 mm. The porosity of such a sample writes $\sigma ={\left(1-\frac{d}{M}\right)}^{2}=0.64$. The TU Delft sample is a sample where a 3D model is reproduced along the test section dimension. The template shape and the geometric characteristics are shown in Fig. 2.

**Table 2:** Sample description (a).

Description of the sample			Measurements	
Sample label	Geometric features	Picture	Pressure	LDV
SCP D5T6e1	Perforation diameter D = 5 mm		X	
	Pitch T = 6 mm			
	Thickness e = 1 mm			
SCP D4T6e1	Perforation diameter D = 4 mm		X	
	Pitch T = 6 mm			
	Thickness e = 1 mm			
SCP D2T3e2	Perforation diameter D = 2 mm		X	X
	Pitch T = 3 mm			
	Thcikness e = 2 mm			
SCP D2T3e1	Perforation diameter D = 2 mm		X	
	Pitch T = 3 mm			
	Thickness e = 1 mm

**Table 3:** Sample description (b).

Description of the sample	Measurements			
Sample label	Geometric features	Picture	Pressure	LDV
HP	No information available		X	
WM	Wire diameter d = 1 mm		X	X
	Mesh size M = 4.5 mm			
WM	No information available		X	
OPAL D2T3.2Phi30e1	Perforation diameter D = 2 mm		X	
	Pitch T = 3 mm			
	Thickness e = 1 mm			
	Angle of the perf. Phi = 30 deg

**Table 4:** Sample description (c).

Description of the sample		Measurements	
Sample label	Picture	Pressure	LDV
TU delft diamond *d*_*c*_ = 2.5 mm		X	
TU delft diamond *d*_*c*_ = 4.5 mm		X	X

### Pressure drop post-processing procedure

The pressure drop coefficient is defined by:

1 $$\xi =\frac{\Delta {P}_{i}}{\frac{\gamma }{2}{P}_{0}{M}^{2}}$$

The mass flow is regulated in the B2A bench and it is assumed that we are in incompressible conditions. The pressure drop coefficient or resistance coefficient writes thus:

2 $$\xi =\frac{\Delta {P}_{s}}{\frac{\gamma }{2}{P}_{0}{M}_{b}^{2}}$$

**Fig. 3:**
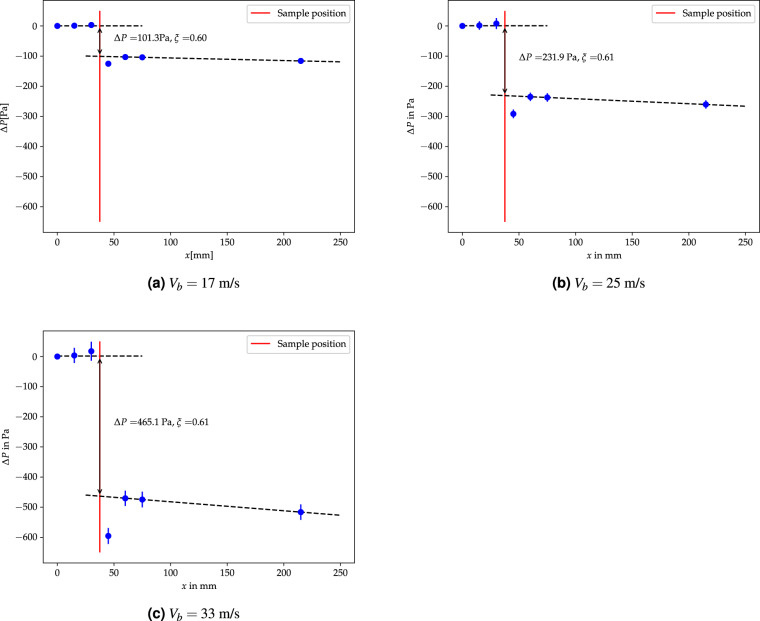
Pressure drop measurement procedure. the differential static pressure measured at each pressure tap with reference to the most upstream one is plotted in blue circle. The pressure drop induced by the sample is assessed from the delta between the two black dashed lines values at the sample position.

Indeed, the total pressure drop Δ*P*_*i*_ is equivalent to the static pressure drop Δ*P*_*s*_. *M*_*b*_ is the bulk Mach number of the test bench derived from the measured mass flow and the temperature is assigned and regulated. The Morgan’s method^15^ is applied to assess the value of Δ*P*_*s*_. This method was also used by Pinker and Herbert^16^. The idea is to use several pressure taps and to retrieve the pressure loss due to the friction loss inside the duct so that only the pressure drop due to the sample is extracted. Figure 3 illustrate the principle of the method applied to the WM sample. For each flow condition, the post-processing proposed by Morgan enables to extract only the pressure drop contribution coming from the sample. This procedure is applied to each sample in order to ensure accurate assessment of the induced pressure drop. By propagating the uncertainties of the different sensors for the calculation of the pressure drop coefficient, we obtain an uncertainty on the value of *ξ* close to 1%.

## Data Records

The full data set is available on zenodo deposit^17^.

Table 5 recalls the flow condition performed during this study and the corresponding Reynolds number based on the B2A test section width.

**Table 5:** Flow conditions at 20 °C.

Mass flow in g/s	50	75	106
Bulk velocity in m/s	17	25	36
Reynolds number based on the B2A dimensions	55000	83000	117000

### Pressure drop results

The pressure drop data record is composed of a csv file called “pressure_drop.csv”. The results are summarized in Table 6.

**Table 6:** Overall pressure drop coefficient *ξ* results.

Mass flow in g/s	50	75	106
Bulk velocity in m/s	17	25	36
WM	0.60	0.61	0.61
SCP D5T6e1	1.21	1.24	1.17
SCP D4T6e1	3.27	3.35	3.22
SCP D2T3e2	2.88	2.60	2.70
SCP D2T3e1	5.03	5.16	5.16
HP	4.13	4.51	4.41
OPAL D2T3.2Phi30e1	13.45	13.99	14.99
Diamond Grids 2.5 mm	110.4	117.5	177.6
Diamond Grids 4.5 mm	62.2	65.6	70.77
WM DLR	56.88	54.54	56.36

### LDV results

The LDV data records are of two types: statistical results on the velocity field and binary results, which include the time evolution of the measured instantaneous velocity (with roughly 200,000 time samples for each measurement point).

Table 7 summarizes the different result file name for the different flow conditions. Each file “Stats_yyy_xxgs_en.dat” is a tecplot format compatible file. The variables can be described as followed:

- “Point”, Number of point which corresponds to the point label for the binary file
- *X*, *Y*, *Z*, Coordinates in mm
- *U*, *W*, Mean value of the velocity in m/s
- *SdevU*, *SdevW*, Standard deviation of the velocity
- *VarU*, *VarW*, Variance of the velocity component
- *SkU*, *SkW*, Skewness of the velocity component
- *FlU*, *FlW*, Flatness of the velocity component
- *UW*, Cross correlated momentum

**Table 7:** Data record file name for statistical results.

Mass flow in g/s	50	75	106
Bulk velocity in m/s	17	25	36
WM	Stats_wmon_50gs_en.dat	Stats_wmon_75gs_en.dat	Stats_wmon_106gs_en.dat
SCP D2T3e2	Stats_scp_50gs_en.dat	Stats_scp_75gs_en.dat	Stats_scp_106gs_en.dat
Diamond Grids 4.5 mm	Stats_tud_50gs_en.dat	Stats_tud_75gs_en.dat	—

Table 8 presents the path for the binary files and which flow conditions they are corresponding to.

**Table 8:** Data record binary path for unsteady velocity results.

Mass flow in g/s	50	75	106
Bulk velocity in m/s	17	25	36
WM	WMON1	WMON2	WMON3
SCP D2T3e2	SCP1	SCP2	SCP3
Diamond Grids 4.5 mm	TUD1	TUD2	—

The binary results are named “INVE0XXX.bin”. The program “PSD_estimation.py” gives some routines to read and calculate the PSD of a given velocity component and a given point. In each data record binary directory, one can find a histogram of the velocity results in order to have a statistic assessment of the measurement uncertainty in repertory called “Histo_Vel”.

**Fig. 4:**
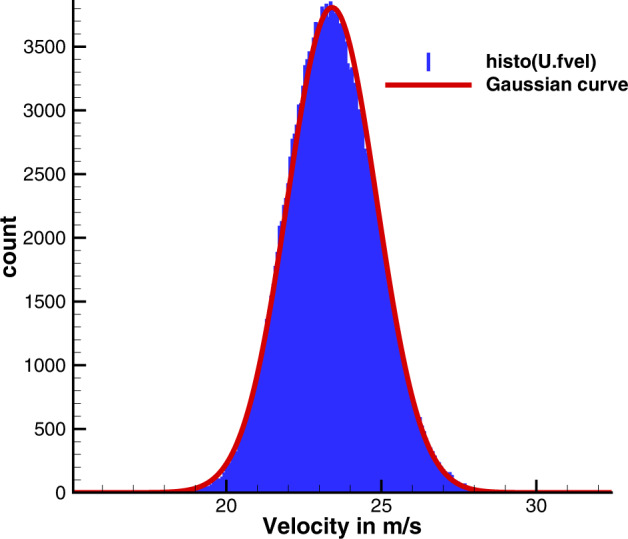
Example of velocity histogram and equivalent Gaussian curve for point #3 of WMON2 (25 m/s), Mean velocity: 23.38 m/s; standard deviation of the velocity: 1.31 m/s.

Figure 4 shows an example of the available results.

## Technical Validation

### Wiremesh pressure drop analysis

For an incompressible flow, *ξ* is only a function of the porosity of the screen and the Reynolds number based on the wire diameter^18^. It has been shown that the pressure resistance coefficient can be approximated as^16^:

3 $$\xi =f\left(R{e}_{d}\right)\frac{1-{\beta }^{2}}{{\beta }^{2}}$$

where *f* is a decaying function of *Re*_*d*_, which is the Reynolds number based on the wire diameter. Roach^19^ suggests to use $f=0.52+66R{e}_{d}^{-4/3}$ while Groth and Johnasson^20^ propose $f=0.4+8.4R{e}_{d}^{-4/5}$. In the *Re*_*d*_ interval 40-10^5^, *f* decays as $R{e}_{d}^{-4/3}$, and asymptotically approaches a constant value in the limit of high *Re*_*d*_.The resistance coefficient can be evaluated by these correlations with a fair degree of accuracy, see Table 9. However, for high accuracy it is necessary to determine the value experimentally for each grid and flow condition, this conclusion can be found in^14,18^.

**Table 9:** Comparison of experimental results and grids correlations for WM sample.

Mass flow in g/s	50	75	106
Bulk velocity in m/s	17	25	33
*ξ WM experimental*	0.600	0.611	0.614
*ξ Groth and Johansson*	0.621	0.609	0.601
*ξ Roach*	0.758	0.754	0.752

### Diamond grids pressure drop analysis

The diamond grids sample can be considered as a flow through a porous media configuration. Table 10 summarizes up the pressure drop coefficient obtained for the 2 types of diamond grids. A good approach to model this type of media is to use the modified Ergun equation^21^:

4 $$\xi =\frac{\alpha e\mu }{(1/2)\rho {U}_{b}\sigma }+\frac{\beta e}{(1/2){\sigma }^{2}}$$

with $\alpha =\frac{A{\left(1-\sigma \right)}^{2}}{\left({\sigma }^{3.6}\ast {D}_{eq}^{2}\right)}$ and $\beta =\frac{B\left(1-\sigma \right)}{{\sigma }^{3.6}{D}_{eq}}$, *e* the sample thickness, here chosen to be equal to *d*_*c*_ (see in diamond sample definition, Fig. 2), *σ* the sample porosity and *D*_*eq*_ a characteristic medium length, here chosen to be equal to *d*_*pore*_ (see in diamond sample definition, Fig. 2). Based on Gupte data base available in^21^, the constants can be chosen as *A* = 132.7 and *B* = 1.291.

**Table 10:** Comparison of experimental results and grids correlations for diamond grids.

Mass flow in g/s	50	75	106
Bulk velocity in m/s	17	25	33
*ξ* experimental *d*_*c*_ = 2.5 mm	110.4	117.5	177.6
*ξ* experimental *d*_*c*_ = 4.5 mm	62.2	65.6	70.77
*ξ* Modified Ergun *d*_*c*_ = 2.5 mm	81.8	80.42	80.33
*ξ* Modified Ergun *d*_*c*_ = 4.5 mm	80.5	79.82	79.61

**Fig. 5:**
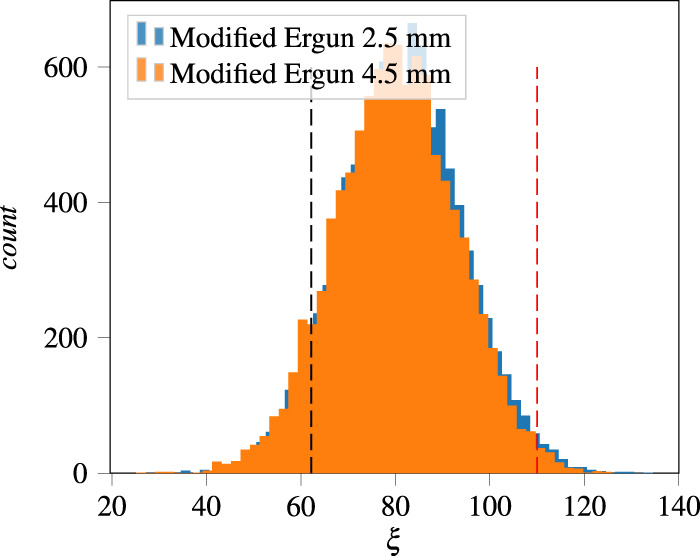
Normal distribution of Modified Ergun model of Diamond samples based on the model parameter distribution^21^. The black and red vertical lines show the experimental value measured for *d*_*c*_ = 4.5 mm and *d*_*c*_ = 2.5 mm, respectively.

Typically, this model should be calibrated on a large amount of samples to give some better results. Presently, in a first approach, the results are fairly good and the order of magnitude is respected even if the decreased of pressure drop between the two grids is not correctly estimated. Figure 5 shows the normal distribution of the modified Ergun model for the diamond samples, based on the geometric parameter of the grids. In Mac Donald *et al*.^21^, the discrepancy between the experimental results and the model fit is available. The same distribution is plotted here in order to quantify this discrepancy while using only the geometric description of the sample. One can notice that the measured results on the diamond samples are in the distribution range which confirms the validity of the modified Ergun model.

### Mean flow LDV results

**Fig. 6:**
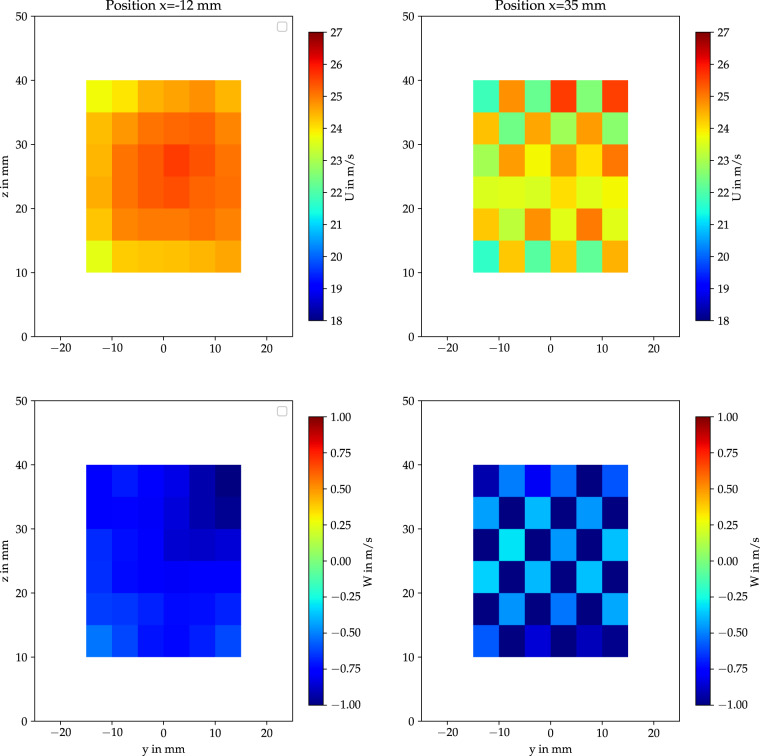
Mean velocity value for plane *x* = −12 mm and *x* = 35 mm. WM sample for a mass flow of 75 g/s.

**Fig. 7:**
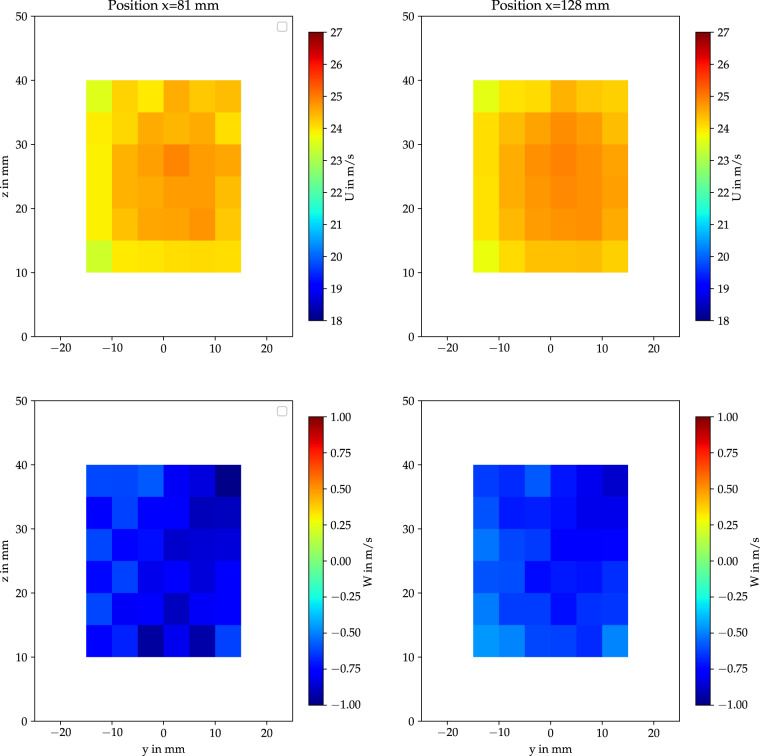
Mean velocity value for plane *x* = 81 mm and *x* = 128 mm. WM sample for a mass flow of 75 g/s.

**Fig. 8:**
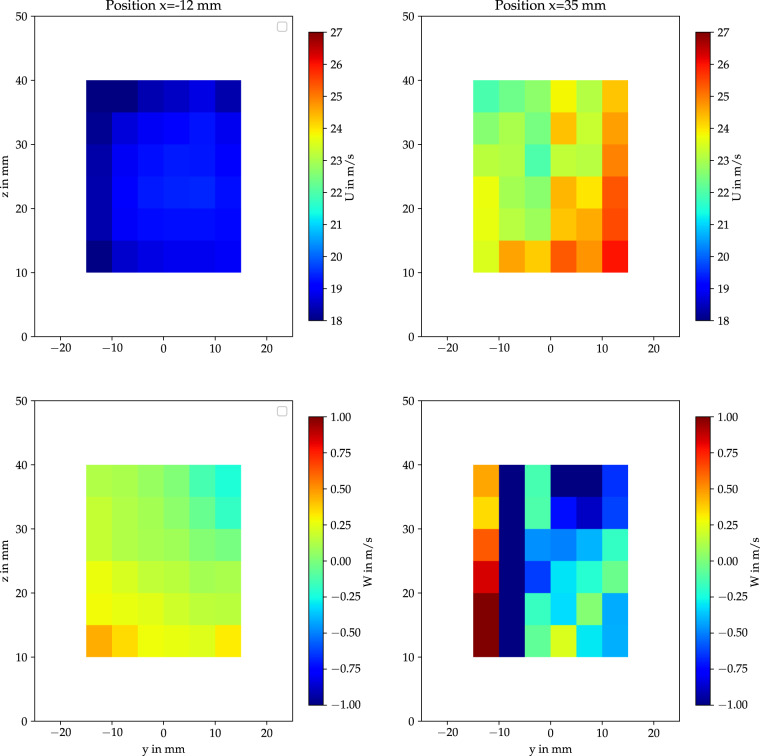
Mean velocity value for plane *x* = −12 mm and and *x* = 35 mm. Diamond 4.5 mm for a mass flow of 75 g/s.

**Fig. 9:**
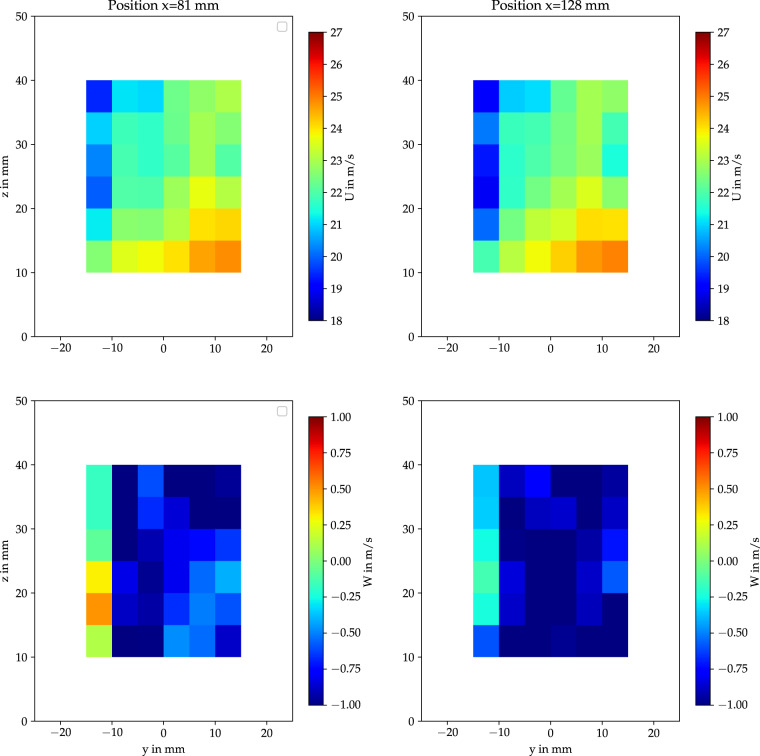
Mean velocity value for plane *x* = 81 mm and *x* = 128 mm. for Diamond 4.5 mm sample for a mass flow of 75 g/s.

**Fig. 10:**
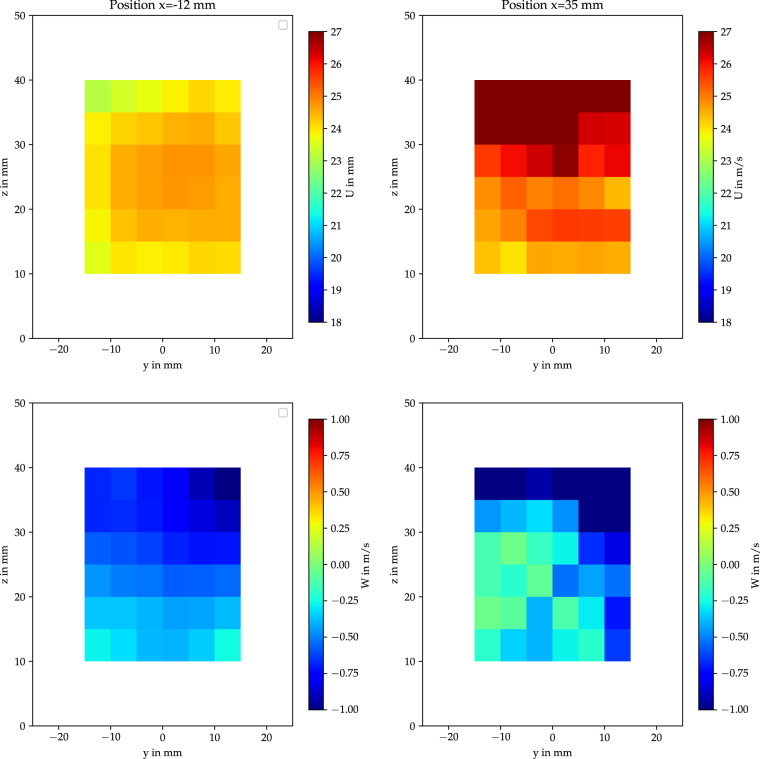
Mean velocity value for plane *x* = −12 mm and and *x* = 35 mm. D2T3e2 sample for a mass flow of 75 g/s.

**Figure 11:**
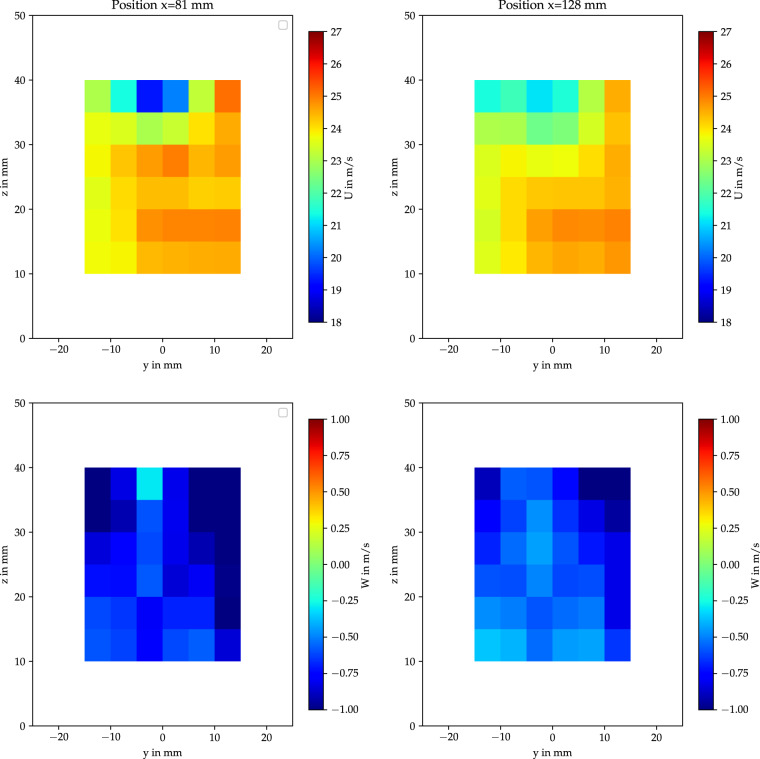
Mean velocity value for plane *x* = 81 mm and *x* = 128 mm. D2T3e2 sample for a mass flow of 75 g/s.

Figures 6–11 present overall all mean flow results. Several remarks should be done on these mean fields. The measurement grids are globally quite coarse (36 points per plane). The spatial resolution is therefore not sufficient to describe the flow structures. However, the main trends can be identified. For the WM sample (Figs. 6, 7), the effect of the wires can be seen on the first downstream position (35 mm). Rapidly, for the next plane position, one can notice that the upstream mean flow conditions seem to be recovered. For the diamond grid (Figs. 8, 9), the incident flow which is measured 12 mm before the sample is highly impacted by a blockage effect due to the pressure loss which has a important impact on the meanflow profile. For the SCP case (Figs. 10, 11) and the diamond grid (Figs. 8, 9), the distance of the measurement planes downstream the samples seems not sufficient to recover a mean condition comparable to the incident flow. The turbulence measurements can give further information about the turbulence decay, this will be detailed in the next section.

### Turbulence characterisation

For each plane, the median value of the turbulence rate is computed:

5 $${T}_{u}={\int }_{y}{\int }_{z}\frac{{u}_{rms}}{U}dydz$$

Downstream of a turbulence generating screen the turbulence intensity decays with a typical power-law decay. It has been found that the appropriate length for the decay is the mesh width M of the screen. The decay can be described by Batchelor and Townsend^22^ law:

6 $${T}_{u}^{2}={\left(\frac{urms}{{U}_{b}}\right)}^{2}=A{\left(\frac{x-{x}_{0}}{M}\right)}^{b}$$

where *x*_0_ is a virtual origin of the screen (that is usually close to the actual position of the screen, in the present study it is the actual position of the sample). The exponent *b* gives the decay rate and the constant A gives the level for a particular screen and Reynolds number. An already identified problem is the large variation of the estimates of these coefficients, which is often explained by an inconsistent determination procedure of the *x*/*M* interval chosen for the curve fit. However, one should not exclude the initial scales generated by the grids as a candidate for the turbulence decay property as argued by several authors. Kurian *et al*.^14^ proposed a systematic approach to characterize the turbulence, by means of energy spectra, characteristic turbulence length scales, energy dissipation, kinetic energy decay rate etc., behind a set of grids with the feature of having roughly the same solidity but different mesh and bar widths.

### Turbulence characterisation of the WM

**Fig. 12:**
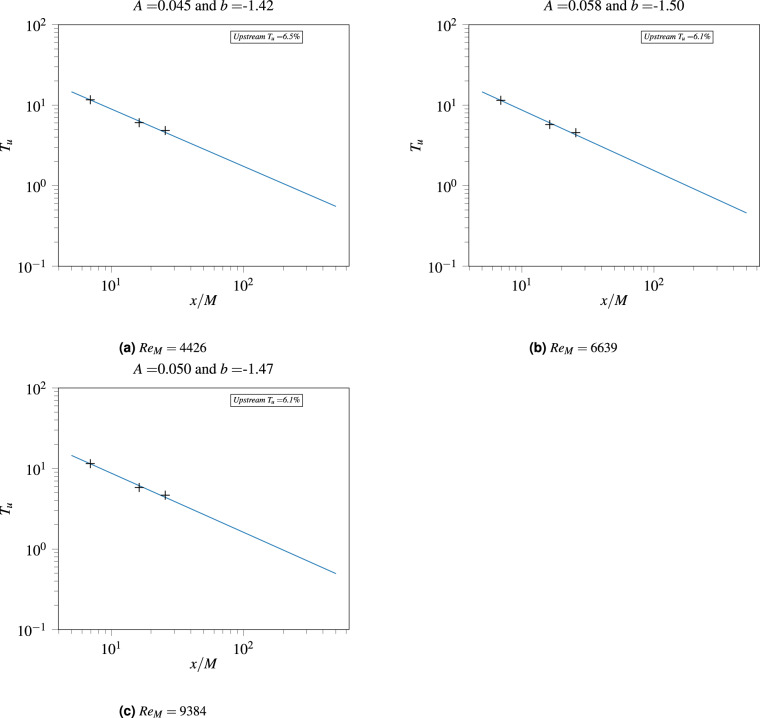
Turbulence decay regression for the WM sample.

**Fig. 13:**
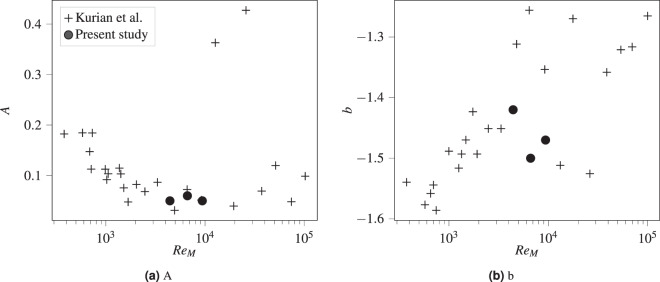
Batchelor and Townsend Turbulence decay for WM and Kurian *et al*.^14^.

The WM sample is typically comparable with Kurian results especially with the mesh LT5 which is almost the same type of mesh; for WM, *M* = 5 mm and *d* = 1 mm. The results for LT5 range from *A* = [0.047; 0.068] and *b* = [−1.45; −1.42] whereas the results for WM appear to range from *A* = [0.047; 0.068] and *b* = [−1.5; −1.42], categorized according to the Reynolds number based on the mesh witdth *M*. Figure 12 shows the turbulence decay parameter regression for the present data set. The turbulence decay is fairly comparable between the present study and Kurian *et al*. results. Figure 13 presents the results from Kurian *et al*. and a comparison with the present results on WM. In Kurian *et al*., comparable samples have been tested and one can notice that the results obtained here with a different set up are really consistent with the cited article.

### Turbulence characterisation of the D2T3e2

**Fig. 14:**
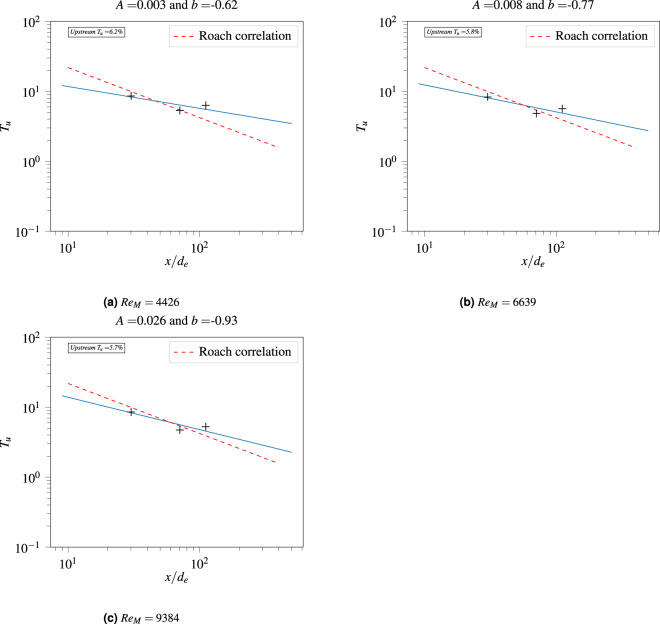
Turbulence decay regression for the D2T3e2 sample.

For the D2T3e2 sample, the *M* value need to be defined. Roach^19^ proposes to use *d*_*e*_ which writes as

7 $$\frac{{d}_{e}}{D}=\frac{1}{{\sigma }^{1/2}}-1$$

as a turbulence length scale with *D* = 2 mm the holes diameter and *σ* = 0.403 the porosity. Figure 14 shows the parameter regression with the present data set.

**Fig. 15:**
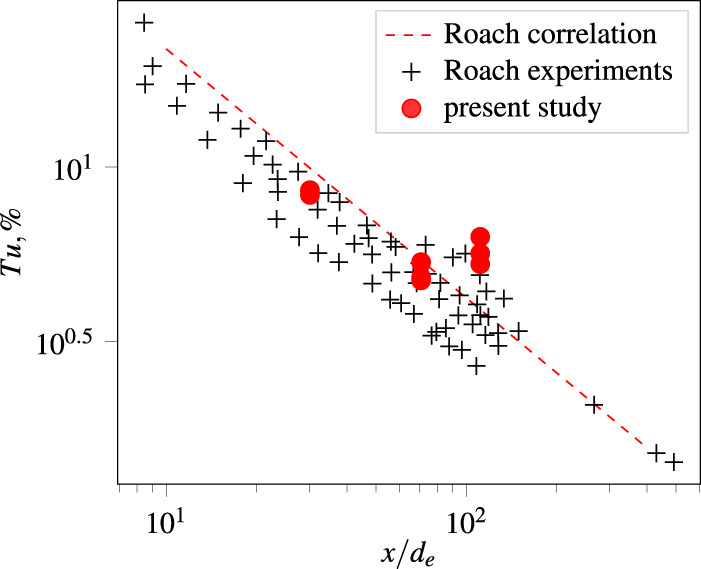
Roach data set and present study comparison.

Roach proposes a correlation

8 $$Tu=1.13{\left(\frac{x}{{d}_{e}}\right)}^{-\frac{5}{7}}$$

which is superimposed to the present results. Note that the last plane gives a turbulence rate which is a little bit higher than the turbulence rate at the previous planes. Nonetheless, the agreement is fairly good with Roach results (see Fig. 15).

### Turbulence characterisation of the diamond sample

**Fig. 16:**
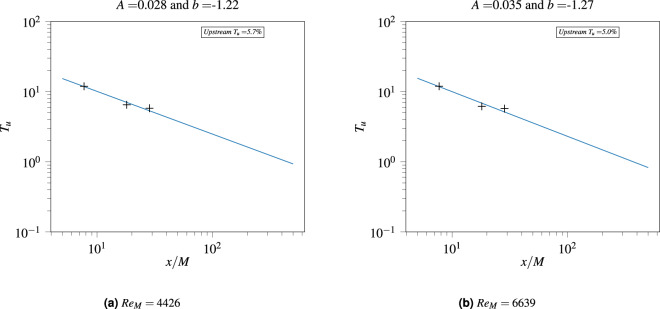
Turbulence decay regression for the Diamond Sample.

**Fig. 17:**
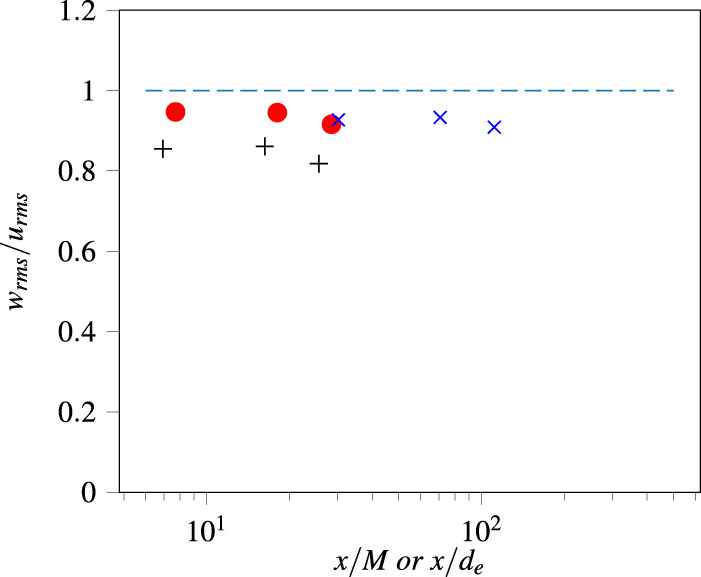
Turbulence anisotropy for WM (black mark), D2T3e2 sample (red mark) and Diamond sample (blue mark), mass flow 75 g/s.

Finally, Fig. 16 shows the turbulence parameter decay using *M* = 4.5 mm as a turbulence length scale for the diamond sample. Figure 17 presents the anisotropy of the turbulence for each samples (median value on each plane). Diamond and D2T3e2 samples seems to be more isotropic than the WM sample.

Several fairing samples have been tested in the B2A test bench in order to create a data base of pressure loss for several mass flow conditions. Three different samples have been studied deeply in term of mean flow and fluctuating turbulence flow components based on LDV measurement enabling canonical configurations that can be reproduced numerically. Preliminary comparisons with correlations available in the literature show a quite good agreement withe the present results.

## Data Availability

In order to read the “*.bin” files, a python code is provided in the zenodo deposit. The program “PSD_estimation.py” gives some routines to read and calculate the PSD of a given velocity component at a given point. Python required version is written as a comment in the python code.
